# PREVALENCE AND RISK FACTORS ASSOCIATED WITH NON-ALCOHOLIC STEATOHEPATITIS IN PATIENTS WITH RHEUMATOID ARTHRITIS ON HYDROXYCHLOROQUINE: A POPULATION-BASED STUDY

**DOI:** 10.1590/S0004-2803.24612024-100

**Published:** 2025-04-04

**Authors:** Antoine BOUSTANY, Somtochukwu ONWUZO, Adejoke JOHNSON, David FARHAT, Mimi NAJJAR, Hadi Khaled Abou ZEID, Chidera N ONWUZO, Mohamad-Noor ABU-HAMMOUR, Rashid ABDEL-RAZEQ, Islam MOHAMED, Barish EREN, Imad ASAAD

**Affiliations:** 1 Division of Gastroenterology, Department of Medicine, University of Florida College of Medicine, Jacksonville, Florida, USA.; 2 Department of Gastroenterology and Hepatology, Allegheny Health Network, Pittsburg, Pennsylvania, USA.; 3 Jacobi Medical Center/North Central Bronx Hospital, Bronx, New York, USA.; 4 Department of Surgery, Johns Hopkins University School of Medicine, Baltimore, Maryland, USA.; 5 The Sydney Kimmel Comprehensive Cancer Center, Johns Hopkins University School of Medicine, Baltimore, Maryland, USA.; 6 Department of Medicine and Medical Sciences, University of Balamand, Koura, Lebanon.; 7 Department of Internal Medicine, SUNY Upstate Medical University, Syracuse, New York, USA.; 8 Department of Medicine, Cleveland Clinic Foundation, Cleveland, Ohio, USA.; 9 Department of Gastroenterology, Firelands Health, Sandusky, OH, USA.

**Keywords:** Nonalcoholic steatohepatitis, rheumatoid arthritis, hydroxychloroquine, Esteato-hepatite não alcoólica, artrite reumatoide, hidroxicloroquina

## Abstract

**Background:**

Non-alcoholic steatohepatitis (NASH) is becoming a leading cause of liver disease in the US, while Rheumatoid arthritis (RA) affects a significant portion of the global population. In recent times, newer drugs have been developed to slow down the progression of RA, one of which is hydroxychloroquine (HCQ). Despite HCQ being linked to slowly progressive transaminitis, its role in the development of NASH remains unclear. Our research fills this gap by examining the prevalence and risk factors of developing NASH in patients with RA on HCQ.

**Methods:**

This retrospective cohort study analyzed 619,350 adult patients diagnosed with RA. Data were sourced from a multicenter database covering over 360 hospitals across 26 healthcare systems in the US from 1999 to September 2022, excluding pregnant individuals. Multivariate regression analysis assessed the risk of NASH, adjusting for confounders including smoking history, male gender, dyslipidemia, hypertension, type 2 diabetes mellitus, obesity, and hydroxychloroquine use. Statistical significance was set at *P*<0.05, with analyses conducted using R version 4.0.2 (R Foundation for Statistical Computing, Vienna, Austria, 2008).

**Results:**

In a cohort of 79.4 million individuals, 619,350 non-pregnant subjects had rheumatoid arthritis, with 3,080 diagnosed with NASH, while 616,270 did not. Patients with NASH displayed a higher prevalence of smoking history, hyperlipidemia, hypertension, type 2 diabetes mellitus, obesity, and HCQ use. Multivariate regression analysis identified increased NASH risk in smokers (OR: 1.24; 95%CI: 1.14-1.36), males (OR: 0.88; 95%CI: 0.81-0.96), individuals with dyslipidemia (OR: 1.34; 95%CI: 1.21-1.47), hypertension (OR: 1.11; 95%CI: 1.00-1.27), type 2 diabetes mellitus (OR: 3.24; 95%CI: 2.98-3.54), obesity (OR: 3.59; 95%CI: 3.31-3.89), and hydroxychloroquine use (OR: 1.79; 95%CI: 1.65-1.94).

**Conclusion:**

RA patients on HCQ showed an increased prevalence and odds of developing NASH, even after adjusting for common confounding factors. This indicates that HCQ may play a role in the development of hepatic steatosis and fibrosis. Clinicians should consider this association to prevent advanced liver disease. Future research should focus on optimal screening for early detection and enhancing patient outcomes.

## HIGHLIGHTS

• The study investigates the relationship between nonalcoholic steatohepatitis (NASH) and rheumatoid arthritis (RA), analyzing the impact of hydroxychloroquine (HCQ) use on the development of NASH.

• HCQ slows the progression of RA; however, its effect on the liver is not yet fully understood.

• This multicenter retrospective cohort study analyzed 619,350 adult patients diagnosed with RA.

• RA patients on HCQ showed an increased prevalence and higher odds of developing NASH. This association should be considered to prevent advanced liver disease.

## RESUMO

**Contexto:**

A esteato-hepatite não alcoólica (EHNA) está se tornando uma das principais causas de doença hepática nos EUA, enquanto a artrite reumatoide (AR) afeta uma parcela significativa da população global. Nos últimos tempos, novos medicamentos foram desenvolvidos para retardar a progressão da AR, um dos quais é a hidroxicloroquina (HCQ). Apesar de a HCQ estar ligada à transamina lentamente progressiva nossa pesquisa preenche essa lacuna examinando a prevalência e os fatores de risco do desenvolvimento de EHNA em pacientes com AR em HCQ.

**Métodos:**

Este estudo de coorte retrospectivo analisou 619.350 pacientes adultos diagnosticados com AR. Os dados foram obtidos de um banco de dados multicêntrico que abrange mais de 360 hospitais em 26 sistemas de saúde nos EUA de 1999 a setembro de 2022, excluindo grávidas. A análise de regressão multivariada avaliou o risco de EHNA, ajustando para fatores de confusão, incluindo história de tabagismo, sexo masculino, dislipidemia, hipertensão, diabetes mellitus tipo 2, obesidade e uso de hidroxicloroquina. A significância estatística foi estabelecida em P<0,05, com análises conduzidas usando o R versão 4.0.2 (R Foundation for Statistical Computing, Viena, Áustria, 2008).

**Resultados:**

Em uma coorte de 79,4 milhões de indivíduos, 619.350 indivíduos não grávidas tinham AR, com 3.080 diagnosticados com EHNA, enquanto 616.270 não. Pacientes com EHNA apresentaram maior prevalência de história de tabagismo, hiperlipidemia, hipertensão, diabetes mellitus tipo 2, obesidade e uso de HCQ. A análise de regressão multivariada identificou aumento do risco de EHNA em fumantes (OR: 1,24; IC95%: 1,14-1,36), homens (OR: 0,88; IC95%: 0,81-0,96), indivíduos com dislipidemia (OR: 1,34; IC95%: 1,21-1,47), hipertensão (OR: 1,11; IC95%: 1,00-1,27), diabetes mellitus tipo 2 (OR: 3,24; IC95%: 2,98-3,54), obesidade (OR: 3,59; IC95%: 3,31-3,89) e uso de hidroxicloroquina (OR: 1,79; IC95%: 1,65-1,94).

**Conclusão:**

Pacientes com AR em HCQ apresentaram aumento da prevalência e chances de desenvolver EHNA, mesmo após ajuste para fatores de confusão comuns. Isso indica que a HCQ pode desempenhar um papel no desenvolvimento de esteatose hepática e fibrose. Os médicos devem considerar essa associação para prevenir doença hepática avançada. Pesquisas futuras devem se concentrar na triagem ideal para detecção precoce e melhorar os resultados dos pacientes.

## INTRODUCTION

Liver diseases, notably non-alcoholic fatty liver disease (NAFLD) and its progressive form, non-alcoholic steatohepatitis (NASH), present significant public health challenges globally^1^. These conditions are closely linked to the rising prevalence of obesity and pose a substantial burden on healthcare systems^2^. NAFLD has become a significant public health concern worldwide, with prevalence estimates ranging from 25% to 35%^2^; exceptionally high prevalence rates have been reported in South American countries (35%), North America (35%), and Europe (30%)^3^. Over the past three decades, there has been a worrisome upward trend, with the global prevalence steadily increasing by 0.7% annually, reaching 37.3% in 2019^4^.

NASH poses a diagnostic challenge due to the requirement for histological confirmation through biopsy, leading to uncertainties surrounding its prevalence. Recent estimates suggest that NASH affects approximately 1.5% to 6.45% of the general population^5^. In the United States alone, an estimated 16.5 million individuals were impacted by NASH in 2015^6^. Furthermore, NASH has emerged as the second most common indication for liver transplantation in the United States, closely trailing alcohol-related liver disease^7^. Moreover, the proportion of NASH-related hepatocellular carcinoma (HCC) cases among patients awaiting liver transplantation has surged significantly by 7.7-fold, escalating from 2.1% to 16.2% in the United States^8^. Up to 10% of patients with NASH will eventually develop cirrhosis, which is the third most common cause of death in NAFLD after cardiovascular disease and cancer^9^. These trends underscore the expanding clinical significance and economic implications of NASH.

NAFLD is characterized by liver fat accumulation exceeding 5% without any association with alcohol consumption^10^. The “two-hit” hypothesis suggests NAFLD begins with steatosis, followed by Mitochondrial dysfunction and decreased ATP synthesis, leading to Reactive oxygen species (ROS) production, endoplasmic reticulum (ER) injury, and lipid peroxidation^9^. This cascade damages cellular components, inducing ballooning, apoptosis, and necrosis. Inflammatory cytokines then trigger inflammation and fibrosis, ultimately culminating in NASH and liver cirrhosis^9^.

Several medical comorbidities, including obesity, metabolic syndrome, type 2 diabetes, insulin resistance, dyslipidemia, and hypertension, are associated with disease progression in NAFLD patients, serving as risk factors for the development of NASH^11^. Notably, a well-documented correlation exists between NAFLD and metabolic syndrome^12^. Patients with this overlap exhibit heightened secretion of fatty acid and triglyceride-rich very low-density lipoproteins (VLDL), attributed to increased fatty acid supply and de novo lipogenesis induced by insulin resistance^11^. These free fatty acids are central to the pathogenesis of NASH. Although the exact mechanisms remain elusive, the development of NAFLD and NASH is believed to result from a complex interplay among these comorbidities, genetic predisposition, environmental factors, and nutritional/behavioral influences^13^. 

In recent years, there has been growing interest in identifying drugs that may contribute to transaminitis and steatohepatitis in susceptible individuals^14^. Hydroxychloroquine (HCQ), a medication commonly used to manage Rheumatoid arthritis (RA), a prevalent chronic inflammatory joint disease affecting around 1% of the world’s population^15^, has received attention in this regard. Despite its widespread use, limited research exists on HCQ’s potential role as an independent risk factor for NAFLD and NASH. To address this gap, we conducted a study to investigate the prevalence of NASH among RA patients receiving HCQ and to assess the associated odds of developing NASH in this patient population.

## METHODS

### Database

Explorys Inc., Cleveland, OH, USA, is a validated multicenter and research platform database of more than 360 hospitals from 26 different healthcare systems across the United States, consisting of data from 1999 to September 2022. Explorys was developed and prospectively maintained by IBM Corporation Watson^16^, including electronic health records (EHR) from more than 60 million unique patients, and provides a broad regional distribution of the United States, representing approximately 15% of the population. It was utilized to construct a retrospective cohort analysis. A Systematized Nomenclature of Medicine-Clinical Terms (SNOMED-CT) hierarchy^17^ was used to select diagnoses, findings, and procedures. Prescription drug orders are mapped into SNOMED and RxNorm^18^. Institutional Review Board (IRB) was not required as source data are de-identified. In the Explorys database, when reporting the number of patients or occurrences of a specific condition, counts are rounded to the nearest 10. Additionally, any count between zero and ten is considered the same, likely to prevent identifying individual patients or cases with very low counts. This practice helps maintain patient confidentiality by obscuring precise counts of small groups or rare occurrences.

The study was conducted in accordance with the Declaration of Helsinki (as revised in 2013). Participating healthcare systems are granted access to the database. The Explorys platform has been validated in multiple fields, including gastroenterology^19^ ^,^ ^20^. 

### Patient selection

The study’s main population comprised adults over 18 years of age diagnosed with rheumatoid arthritis, with a subsequent subgroup analysis centering on patients diagnosed with NASH. Control subjects without a diagnosis of NASH were selected from adult patients, ensuring comparability through matching for key demographic variables. Pregnant individuals were excluded from the analytical cohort.

### Statistical analysis

Patients who developed NASH were compared to those who did not. A multivariate regression analysis was performed to account for potential confounders, including smoking history, male gender, individuals with dyslipidemia, hypertension, type 2 diabetes mellitus, obesity, and those who have been using hydroxychloroquine. A two-sided *P* value <0.05 was considered statistically significant, and all statistical analyses were performed using R version 4.0.2 (R Foundation for Statistical Computing, Vienna, Austria, 2008).

## RESULTS

### Descriptive epidemiology

A total of 79,428,638 individuals aged over 18 years underwent comprehensive screening within the database, revealing 619,350 non-pregnant subjects diagnosed with rheumatoid arthritis. Among this cohort, 3,080 individuals were additionally diagnosed with NASH, while 616,270 did not present with this condition. The baseline characteristics of our cohort are displayed in Table 1. Smoking history (21.42%), hyperlipidemia (78.57%), hypertension (83.76%), type 2 diabetes mellitus (64.61%), obesity (66.23%), and individuals who have been using hydroxychloroquine (28.25%) were more common in patients with NASH compared to the one without the disease.

**TABLE 1:** Baseline characteristics of patients with NASH and control

Baseline characteristics	RA with NASH (%) (N=3,080)	RA without NASH (%)(N=616,270)
Sex		
Male	700 (22.72)	159,850 (25.93)
Co-morbidities		
Smoker	660 (21.42)	97,550 (15.82)
Hyperlipidemia	2,420 (78.057)	333,610 (54.13)
Hypertension	2,580 (83.76)	388,480 (63.03)
T2DM	1,990 (64.61)	161,370 (26.18)
Obesity	2,040 (66.23)	155,730 (25.26)
Medication History		
Hydroxychloroquine	870 (28.24)	109,900 (17.83)

NASH: non-alcoholic steatohepatitis; T2DM: type 2 diabetes mellitus.

### Risk and predictors of NASH in patients using a multivariate regression analysis

**FIGURE 1:**
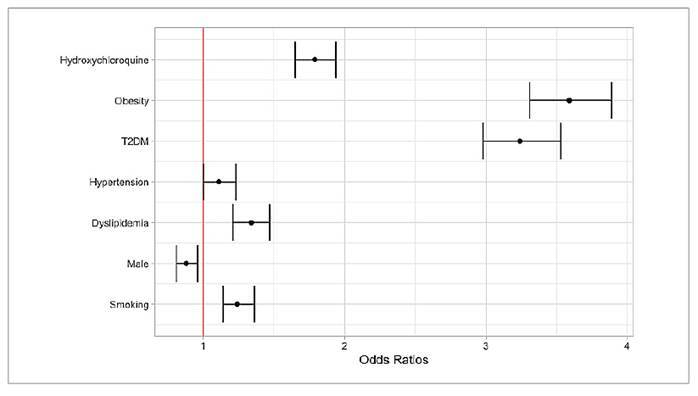
Forest plot for risk of developing NASH compared to control.

NASH: non-alcoholic steatohepatitis; T2DM: type 2 diabetes mellitus.

A multivariate regression analysis was performed to adjust for confounding variables (Figure 1).

The risk of NASH was increased in patients in smokers (OR: 1.24; 95%CI: 1.14-1.36), male (OR: 0.88; 95%CI: 0.81-0.96), individuals with dyslipidemia (OR: 1.34; 95%CI: 1.21-1.47), hypertension (OR: 1.11; 95%CI: 1.00-1.27), type 2 diabetes mellitus (OR: 3.24; 95%CI: 2.98-3.54), obesity (OR: 3.59; 95%CI: 3.31-3.89), and individuals who have been using hydroxychloroquine (OR: 1.79; 95%CI: 1.65-1.94) as seen in Figure 1.

## DISCUSSION

In this population-based study investigating risk factors associated with NASH in rheumatoid arthritis patients, we observed a notable increase in the odds of developing NASH among individuals with obesity, type 2 diabetes mellitus, dyslipidemia, and hypertension. These comorbidities, except for hydroxychloroquine therapy, are established contributors to the pathogenesis of NAFLD and subsequent NASH, representing well-established risk factors^11^.

The association between HCQ and NASH remains controversial in the current literature. While some studies suggest potential protective effects of HCQ in obese patients^21^ ^,^ ^22^, possibly through downregulating adipogenesis to decrease hepatic steatosis^21^, others indicate possible liver toxicity due to reactive metabolites and oxidative stress^23^ ^,^ ^24^. A retrospective review of 1,285 adult rheumatoid arthritis patients using HCQ at a tertiary academic rheumatology practice found a 24% decrease in incident of NAFLD^25^. However, it’s essential to note that this finding was not statistically significant, with an adjusted odds ratio of 0.76 (95%CI: 0.22-2.6, *P*=0.24)^25^. However, our findings revealed a significant correlation between the use of HCQ and the development of NASH, meriting further investigation and clinical consideration. 

HCQ undergoes hepatic metabolism primarily by the hepatic cytochrome P-450 system^26^. Emerging literature suggests a potential for HCQ-induced liver toxicity attributed to various factors, including reactive metabolites, oxidative stress, and inflammatory processes^27^. Experimental studies administering chloroquine to rats have demonstrated increased activity of liver myeloperoxidase enzyme, lipid peroxidation, protein carbonylation, and depletion of tissue glutathione, collectively leading to oxidative stress induction^28^. Oxidative stress is recognized as a pivotal mechanism in liver damage, particularly in the progression from simple hepatic steatosis to more severe conditions such as NASH^29^. 

Furthermore, a separate study examining the effects of HCQ on vital organ function in male albino rats revealed Kupffer cell hyperplasia and occasional hepatocyte dysplasia^30^; Kupffer cell hyperplasia typically signifies ongoing hepatic inflammation, with activated macrophages playing a critical role in the pathogenesis of NAFLD, including its progression to NASH and fibrosis^31^. A recent study highlighted the pivotal role of Kupffer cells in progressing non-alcoholic steatosis to steatohepatitis and fibrosis^32^. The study identified chitotriosidase (CHIT), an enzyme expressed exclusively in Kupffer cells, as significantly elevated in liver biopsies from NASH patients compared to those with simple steatosis^32^. CHIT overexpression influenced hepatic stellate cell activation, indicating its involvement in fibrosis development^32^. Given that HCQ has been linked to Kupffer cell hyperplasia, it raises questions about whether increased CHIT production in RA patients using HCQ could lead to molecular changes in the liver, potentially contributing to the development of NASH and liver cirrhosis. Further investigation into these molecular mechanisms is warranted to understand the full implications for HCQ therapy patients. 

A recent 2022 study investigated how HCQ affects macrophages, essential for managing lipids and forming foam cells, to assess its potential in preventing dyslipidemia and atherosclerotic cardiovascular disease (ASCVD) in RA patients^33^. Results showed HCQ did not significantly change lipid accumulation in macrophages, raising uncertainty about its impact on heart health and ASCVD risk in RA^33^. This uncertainty extends to the development of NASH in RA patients due to similar risks of cardiac events.

The extensive screening and inclusion of a large patient cohort in our study enhance the generalizability and reproducibility of our findings. Moreover, the higher odds of developing NASH in patients with RA who are using hydroxychloroquine after adjusting for confounding variables mitigates the risk of confounding bias. However, notable limitations warrant consideration. Our study did not address the potential influence of HCQ dosage and duration despite its well-established dose-dependent toxicities. Additionally, we did not account for concurrent medications that may interact with HCQ metabolism or independently induce hepatotoxic effects. Furthermore, the disease status of rheumatoid arthritis patients may influence NASH development and should be factored into future investigations. Since the diagnosis are based on ICD codes, information on how the diagnosis of steatohepatitis was made could not be retrieved. Alcohol use was not excluded or control for to ensure our population was representative and reflective of the real-world population. Lastly, since the ICD code used was for “non-alcoholic steatohepatitis”, we were unable to adopt the recently published new nomenclature^34^.

## CONCLUSION

Our study establishes a significant correlation between HCQ use and NASH development, prompting further investigation into HCQ’s hepatic metabolism and potential liver damage induction. Clinicians should be aware of this association to prevent advanced liver disease, emphasizing the need for additional research to establish optimal screening protocols for early disease detection and improved patient outcomes.

## References

[1] Mitra S, De A, Chowdhury A (2020). Epidemiology of non-alcoholic and alcoholic fatty liver
diseases. Transl Gastroenterol Hepatol.

[2] Huang DQ, El-Serag HB, Loomba R (2021). Global epidemiology of NAFLD-related HCC: trends, predictions,
risk factors and prevention. Nat Rev Gastroenterol Hepatol.

[3] Le MH, Yeo YH, Li X, Li J, Zou B, Wu Y (2022). 2019 Global NAFLD Prevalence: A Systematic Review and
Meta-analysis. Clin Gastroenterol Hepatol.

[4] Teng ML, Ng CH, Huang DQ, Chan KE, Tan DJ, Lim WH (2023). Global incidence and prevalence of nonalcoholic fatty liver
disease. Clin Mol Hepatol.

[5] Younossi ZM, Koenig AB, Abdelatif D, Fazel Y, Henry L, Wymer M (2016). Global epidemiology of nonalcoholic fatty liver
disease-Meta-analytic assessment of prevalence, incidence, and
outcomes. Hepatology.

[6] Estes C, Razavi H, Loomba R, Younossi Z, Sanyal AJ (2018). Modeling the epidemic of nonalcoholic fatty liver disease
demonstrates an exponential increase in burden of disease. Hepatology.

[7] Noureddin M, Vipani A, Bresee C, Todo T, Kim IK, Alkhouri N (2018). NASH Leading Cause of Liver Transplant in Women: Updated Analysis
of Indications For Liver Transplant and Ethnic and Gender
Variances. Am J Gastroenterol.

[8] Younossi Z, Stepanova M, Ong JP, Jacobson IM, Bugianesi E, Duseja A (2019). Nonalcoholic steatohepatitis is the fastest growing cause of
hepatocellular carcinoma in liver transplant candidates. Clin Gastroenterol Hepatol.

[9] Sharma B, John S (2024). StatPearls.

[10] Wang TY, Wang RF, Bu ZY, Targher G, Byrne CD, Sun DQ (2022). Association of metabolic dysfunction-associated fatty liver
disease with kidney disease. Nat Rev Nephrol.

[11] Cotter TG, Rinella M (2020). Nonalcoholic Fatty Liver Disease 2020: The State of the
Disease. Gastroenterology.

[12] Marchesini G, Bugianesi E, Forlani G, Cerrelli F, Lenzi M, Manini R (2003). Nonalcoholic fatty liver, steatohepatitis, and the metabolic
syndrome. Hepatology.

[13] Juanola O, Martínez-López S, Francés R, Gómez-Hurtado I (2021). Non-Alcoholic Fatty Liver Disease: Metabolic, Genetic, Epigenetic
and Environmental Risk Factors. Int J Environ Res Public Health.

[14] Satapathy SK, Kuwajima V, Nadelson J, Atiq O, Sanyal AJ (2015). Drug-induced fatty liver disease: An overview of pathogenesis and
management. Annals of Hepatology.

[15] Alivernini S, Tolusso B, Petricca L, Ferraccioli G, Gremese E (2019). Elsevier eBooks.

[16] Health IBM Corporation The IBM Explorys Platform: liberate your healthcare data.

[17] US National Library of Medicine Unified Medical Language System
(UMLS) (2021). Systematized Nomenclature of Medicine- Clinical Terms (SNOMED
CT).

[18] Nelson SJ, Zeng K, Kilbourne J, Powell T, Moore R (2011). Normalized names for clinical drugs: RxNorm at 6
years. J Am Med Inform Assoc.

[19] Boustany A (2023). Epidemiology and risk of colorectal cancer in patients with a
history of Helicobacter pylori infection: a population-based
study. Ann Gastroenterol.

[20] Boustany A, Mardelli M, Onwuzo S, Coleman A, Zeid HKA, Almomani A (2022). Increased Risk of Central Retinal Vein Occlusion in Patients with
Inflammatory Bowel Disease: A Population-Based Study. J Ophtalmol.

[21] Qiao X, Zhou ZC, Niu R, Su YT, Sun Y, Liu HL (2019). Hydroxychloroquine Improves Obesity-Associated Insulin Resistance
and Hepatic Steatosis by Regulating Lipid Metabolism. Front Pharmacol.

[22] Association of Hydroxychloroquine (2020). Use with Development of Non-Alcoholic Fatty Liver Disease in Rheumatoid
Arthritis - ACR Meeting Abstracts.

[23] Abdel Galil SM (2015). Hydroxychloroquine-induced toxic hepatitis in a patient with
systemic lupus erythematosus: a case report. Lupus.

[24] Giner Galvañ V, Oltra MR, Rueda D, Esteban MJ, Redón J (2007). Severe acute hepatitis related to hydroxychloroquine in a woman
with mixed connective tissue disease. Clin Rheumatol.

[25] Association of Hydroxychloroquine (2020). Use with Development of Non-Alcoholic Fatty Liver Disease in Rheumatoid
Arthritis - ACR Meeting Abstracts.

[26] Stokkermans TJ, Falkowitz DM, Trichonas G (2024). Chloroquine and hydroxychloroquine toxicity.

[27] Falcão MB, Pamplona de Góes Cavalcanti L, Filgueiras NM, Antunes de Brito CA (2020). Case Report: Hepatotoxicity Associated with the Use of
Hydroxychloroquine in a Patient with COVID-19. Am J Trop Med Hyg.

[28] Niknahad H, Heidari R, Firuzi R, Abazari F, Ramezani M, Azarpira N (2016). Concurrent Inflammation Augments Antimalarial Drugs-Induced Liver
Injury in Rats. Adv Pharm Bull.

[29] Martín-Fernández M, Arroyo V, Carnicero C, Sigüenza R, Busta R, Mora N (2022). Role of Oxidative Stress and Lipid Peroxidation in the
Pathophysiology of NAFLD. Antioxidants.

[30] Alruw~aili M, Jarrar B, Jarrar Q, Alruwaili M, Goh KW, Moshawih S (2023). Hydroxychloroquine Toxicity in the Vital Organs of the Body: In
Vivo Study. Front Biosci.

[31] Kazankov K, Barrera F, Møller HJ, Rosso C, Bugianesi E, David E (2016). The macrophage activation marker sCD163 is associated with
morphological disease stages in patients with non-alcoholic fatty liver
disease. Liver Int.

[32] Malaguarnera L, Di Rosa M, Zambito AM, dell’Ombra N, Di Marco R, Malaguarnera M (2006). Potential role of chitotriosidase gene in nonalcoholic fatty
liver disease evolution. Am J Gastroenterol.

[33] Ahmed S, Konig J, Kasselman LJ, Renna HA, De Leon J, Carsons SE (2022). Hydroxychloroquine Effects on THP-1 Macrophage Cholesterol
Handling: Cell Culture Studies Corresponding to the TARGET
Cardiovascular. Trial. Medicina (Kaunas).

[34] Eslam M, Sanyal AJ, George J, International Consensus Panel (2020). MAFLD: A Consensus-Driven Proposed Nomenclature for Metabolic
Associated Fatty Liver Disease. Gastroenterology.

